# Novel volumetric modulated arc therapy approach for lattice radiation therapy for bulky liver tumors

**DOI:** 10.3389/fonc.2025.1680342

**Published:** 2025-10-31

**Authors:** Christine V. Chung, Saurabh S. Nair, Meena S. Khan, Callistus I. Nguyen, Rachael M. Martin-Paulpeter, Ethan B. Ludmir, Laurence E. Court, Joshua S. Niedzielski

**Affiliations:** ^1^ Department of Radiation Physics, The University of Texas MD Anderson Cancer Center, Houston, TX, United States; ^2^ Department of Gastrointestinal Radiation Oncology, The University of Texas MD Anderson Cancer Center, Houston, TX, United States

**Keywords:** spatially fractionated radiation therapy, lattice radiation therapy, VMAT, RapidArc Dynamic, liver cancer, hepatic cancer, bulky tumors, peak-to-valley dose ratio

## Abstract

**Purpose:**

Lattice radiation therapy (LRT) is a type of spatially fractionated radiation therapy that has emerged as an effective treatment approach for bulky solid tumors. RapidArc Dynamic (RAD) is a novel beam delivery approach that may be advantageous for LRT. The purpose of this *in silico* study was to evaluate and compare a novel RAD-based LRT approach (RAD-LRT) with conventional volumetric modulated arc therapy (VMAT)-based LRT (VMAT-LRT).

**Methods:**

Twenty patients with bulky liver tumors treated with RT were retrospectively identified. VMAT-LRT and RAD-LRT plans were generated for all patients. Lattice spheres were placed in a standardized hexagonal pattern with alternating high-dose spheres (vertex tumor volume high [VTVH], analogous to the peak dose) and low-dose control spheres (vertex tumor volume low [VTVL], analogous to the valley dose). Gross tumor volumes (GTVs)<1,000 cm^3^ and GTVs ≥1,000 cm^3^ were planned with 1.0-cm-diameter spheres (n=10) and 1.5-cm-diameter sphere (n=10), respectively. A prescription dose of 20 Gy to 80% of the VTVH was utilized. LRT dose metrics (e.g., peak-to-valley dose ratios, VTVH D80, VTVL D_mean_) were calculated and were compared using paired Wilcoxon sign-ranked test. Planning efficiency was assessed by evaluating planning structures, planning time, and number of treatment fields.

**Results:**

For all 20 cases, RAD-LRT achieved superior plan quality than VMAT-LRT, indicated by similar prescription dose coverage (group mean, VTVH D80: 20.40 Gy for VMAT-LRT, 20.50 Gy for RAD-LRT) but significantly lower valley dose (group mean, VTVL mean dose: 3.40 Gy for VMAT-LRT, 2.20 Gy for RAD-LRT, p<0.0001). Compared to VMAT-LRT, RAD-LRT required fewer planning structures (mean ± SD, 9 ± 1 for VMAT-LRT, 4 ± 1 for RAD-LRT), less planning time (26 ± 8 min for VMAT-LRT, 18 ± 11 min for RAD-LRT), and fewer treatment beams (5 ± 1 arcs for VMAT-LRT, 1 arc with 4 ± 1 static ports for RAD-LRT). RAD-LRT also had significantly higher peak-to-valley dose ratios (group mean, VTVH/VTVL D90 ratio: 8.92 for VMAT-LRT, 18.20 for RAD-LRT, p<0.0001).

**Conclusion:**

RAD may offer a unique approach to Lattice RT. RAD-LRT generated high quality plans with notable treatment planning efficiency, allowing for creation of quality plans without extensive planning time and LRT expertise.

## 1 Introduction

The evolution of radiation therapy, spurred by recent advances in technology and automation, continues to drive the exploration of innovative dose delivery strategies and treatment approaches. The management of large, bulky tumors remains complex, where maximizing tumor control and minimizing normal tissue toxicity can often make treatment planning both technically demanding and time intensive. Extensive liver tumors are a particular challenge due to proximity to organs at risk (OARs) (1–5). The pursuit of more effective and less toxic radiation therapy for large tumors has led to the exploration of spatially fractionated radiation therapy (SFRT) dose delivery techniques as a promising strategy for tumor debulking without increasing dose to normal tissues (5, 6). Although SFRT has existed for nearly a century, it was not until 2010 that the concept was translated into a 3D approach, known as lattice radiotherapy (LRT) (6, 7).

LRT is characterized by its lattice-like dose pattern of alternating high-dose peaks and low-dose valleys. High-dose peaks not only destroy tumor cells but also have been shown to elicit tumor-specific immunogenic responses (7, 8). Adjacent low-dose valleys appear to serve as a facilitator of this latter effect by preserving tumor vasculature, thereby allowing for perfusion of immunogenic factors (7, 9, 10). Furthermore, the bystander effect allows for a synergy with the immunomodulatory effects of SFRT, as the high-dose peaks induce sufficient cancer cell killing to achieve tumor control, despite not irradiating the entire GTV to a large homogenous dose of radiation that would otherwise suppress or kill host immune cells (11–15).

Often, the most challenging part of treatment planning for LRT is achieving sufficiently low valleys (i.e., often 20–25% of the prescription dose) without compromising the prescription goal (7, 16, 17). LRT is typically delivered as a volumetric modulated arc therapy (VMAT) treatment. While VMAT offers higher conformality and lower delivery time than intensity-modulated RT (IMRT), VMAT induces a low-dose wash of the entire treatment field, which is a drawback in SFRT if a minimal valley dose is desired.

RapidArc Dynamic (RAD) is a novel treatment approach that has the ability to combine the benefits of VMAT and IMRT by incorporating static angles within the treatment arc, allowing for enhanced modulation at strategic angles. Moreover, RAD allows for a dynamic collimator that is optimized alongside the treatment plan, which can increase conformality without requiring multiple arcs. These features suggest a potential advantage of RAD-based LRT (RAD-LRT) over VMAT-based LRT (VMAT-LRT). We investigated the feasibility and dosimetric implications of employing RAD for the delivery of LRT. Specifically, we compared RAD-LRT with VMAT-LRT in terms of fulfillment of plan objectives, normal tissue sparing, and the efficiency of treatment planning.

## 2 Materials and methods

Computed tomography (CT) scans (2.5 mm slice thickness) from 20 patients were utilized for this planning study and were selected to include a large range of unresected, bulky hepatic tumor volumes. All patients had been treated with conventional external beam RT during 2020–2024. Gross tumor volumes (GTVs) ranged from 552 cm^3^ to 2,578 cm^3^ and were used to divide the cases into 2 subgroups: those with GTV less than 1,000 cm^3^ (GTV_<1000_; n=10) and those with GTV 1,000 cm^3^ or larger (GTV_≥1000_; n=10), see **Table 1** for details per case. A dosimetrist and medical physicist, both with multiple years of clinical experience, generated VMAT-LRT and RAD-LRT plans for each case, and dosimetric analysis and comparison was performed. All procedures were performed in compliance with the Declaration of Helsinki and institutional guidelines, and this study was approved by the Institutional Review Board of The University of Texas MD Anderson Cancer Center (IRB number RCR03-400), which waived the requirement for informed consent.

**Table 1:** Case details.

Case no.	V_GTV_ [cm^3^]	V_GTV_ V_Liver_	V_VTVH_ V_GTV_	Sphere diameter [cm]	No. of VTVH spheres	No. of VTVL spheres	No. of sphere layers	VMAT No. of		RAD No. of	
								Arcs	MUs	Arcs	MUs
1	2578	85%	1.20%	1.5	18	22	5	5	14681	1 + 5SP	14878
2	2202	59%	1.10%	1.5	15	17	5	5	16603	1 + 4SP	14161
3	1938	74%	0.80%	1.5	9	15	5	4	12632	1 + 5SP	9509
4	1872	44%	1.00%	1.5	11	15	3	5	13324	1 + 5SP	16374
5	1584	40%	0.60%	1.5	6	10	3	5	10831	1 + 4SP	11327
6	1549	35%	0.70%	1.5	7	15	3	5	9526	1 + 5SP	13349
7	1434	58%	0.90%	1.5	8	14	3	5	12510	1 + 4SP	15007
8	1271	47%	0.60%	1.5	5	12	2	5	8716	1 + 4SP	13067
9	1196	57%	0.80%	1.5	6	10	3	5	9592	1 + 4SP	11889
10	1066	46%	0.60%	1.5	4	6	3	5	7927	1 + 5SP	11486
11	993	33%	0.60%	1	13	17	3	5	7927	1 + 5SP	16446
12	936	41%	0.60%	1	10	12	3	4	11824	1 + 5SP	13035
13	880	39%	0.50%	1	10	11	2	4	11824	1 + 6SP	12824
14	786	35%	0.50%	1	8	18	3	5	12081	1 + 4SP	10931
15	733	25%	0.60%	1	9	12	3	4	13626	1 + 5SP	15741
16	713	36%	0.40%	1	6	12	3	4	8851	1 + 4SP	9620
17	660	26%	0.40%	1	5	5	2	5	10544	1 + 3SP	12701
18	635	36%	0.40%	1	4	8	2	5	11247	1 + 4SP	14772
19	632	25%	0.70%	1	10	15	3	5	13320	1 + 3SP	12427
20	552	16%	0.40%	1	6	8	2	4	7936	1 + 4SP	11230

No., number; V_GTV_, volume of GTV; V_GTV_/V_Liver_, percent of total liver volume that is GTV; V_VTVH_/V_GTV,_ proportion of GTV that is VTVH; VTVH, vertex tumor volume high; VTVL, vertex tumor volume low; VMAT, volumetric modulated arc therapy; RAD, rapid arc dynamic; SP, static ports.

### 2.1 Lattice setup

**Figure 1:**
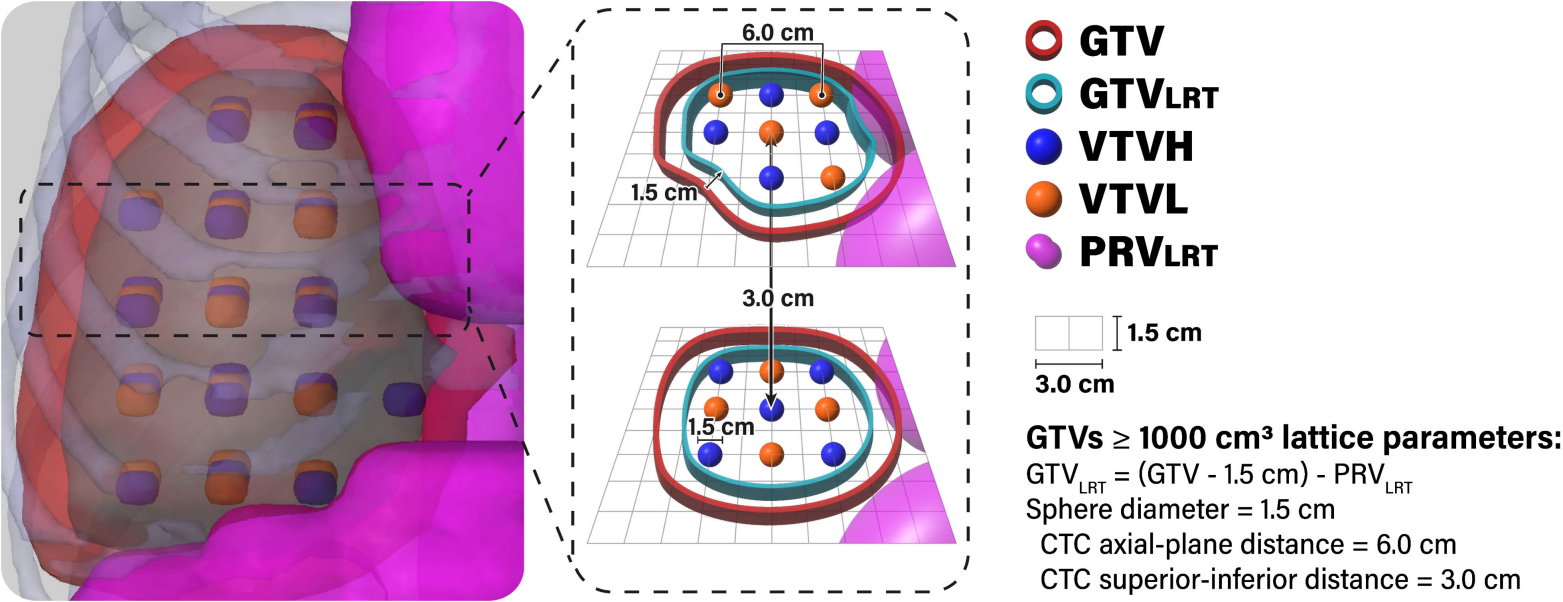
Lattice arrangement for GTV _≥1000_ cases. The GTV_LRT_ was created by contracting the GTV by 1.5 cm and subtracting the PRV_LRT_ (1.5-cm expansion of gastrointestinal luminal contours). Spheres alternated between high-dose spheres (VTVH) and low-dose spheres (VTVL) in all directions. VTVH and VTVL were both 1.5 cm in diameter and spaced with 6.0-cm CTC distance in the axial plane. An in-house custom script was used to automatically generate and place spheres and to remove VTVH outside of the GTV_LRT_ and VTVL outside of the GTV.

Seventeen patients were originally treated with breath-hold motion management, with the remaining 3 receiving free-breathing treatments using the average CT scan generated from 4DCT simulation datasets (in which case, the GTV was delineated using the maximum intensity projection scan). Original clinical OAR structures and GTVs were utilized for planning and evaluation. As these patients were originally treated with conventional RT, all LRT-related structures, including planning targets, were newly generated for this planning study. The majority of LRT contours were generated automatically using an in-house custom script designed to standardize the sphere placements and planning approach. To ensure a conformal distribution, lattice spheres were placed in an alternating pattern with alternating high-dose spheres (vertex tumor volume high [VTVH], analogous to the peak dose) and low-dose control spheres (vertex tumor volume low [VTVL], analogous to the valley dose) in 3D space, a method adapted from the LITE SABR M1 protocol (18). GTV_<1000_ cases were configured with 1.0-cm-diameter spheres with 4.0-cm sphere center-to-center (CTC) distance in the axial plane and 3.0-cm CTC distance in the superior-inferior direction. GTV_≥1000_ cases utilized 1.5-cm-diameter spheres spaced with 6.0-cm center-to-center (CTC) distance in the axial plane and 3.0-cm CTC distance in the superior-inferior direction (19). The lattice arrangement for GTV_≥1000_ cases is diagrammed in **Figure 1**.

In order to determine the allowable region for placing VTVH spheres, the in-house custom script first generates a planning OAR volume (PRV_LRT_) through concatenation of all gastrointestinal luminal contours with an expansion of 1.0 cm for GTV_<1000_ cases and 1.5 cm for GTV_≥1000_ cases (7, 20, 21). Then, it creates the GTV_LRT_ by contracting the clinical GTV by 1.0 cm for GTV_<1000_ cases and 1.5 cm for GTV_≥1000_ cases and subtracting the PRV_LRT_ (**Figure 1**) (7, 22, 23). The script automatically removes whole VTVH spheres outside of the GTV_LRT_, enabling optimal sparing of OARs and minimizing dose to the periphery of the GTV (7, 20–22). Whole VTVL spheres outside of the GTV are also removed.

### 2.2 Treatment planning

#### 2.2.1 VMAT-LRT

**Figure 2:**
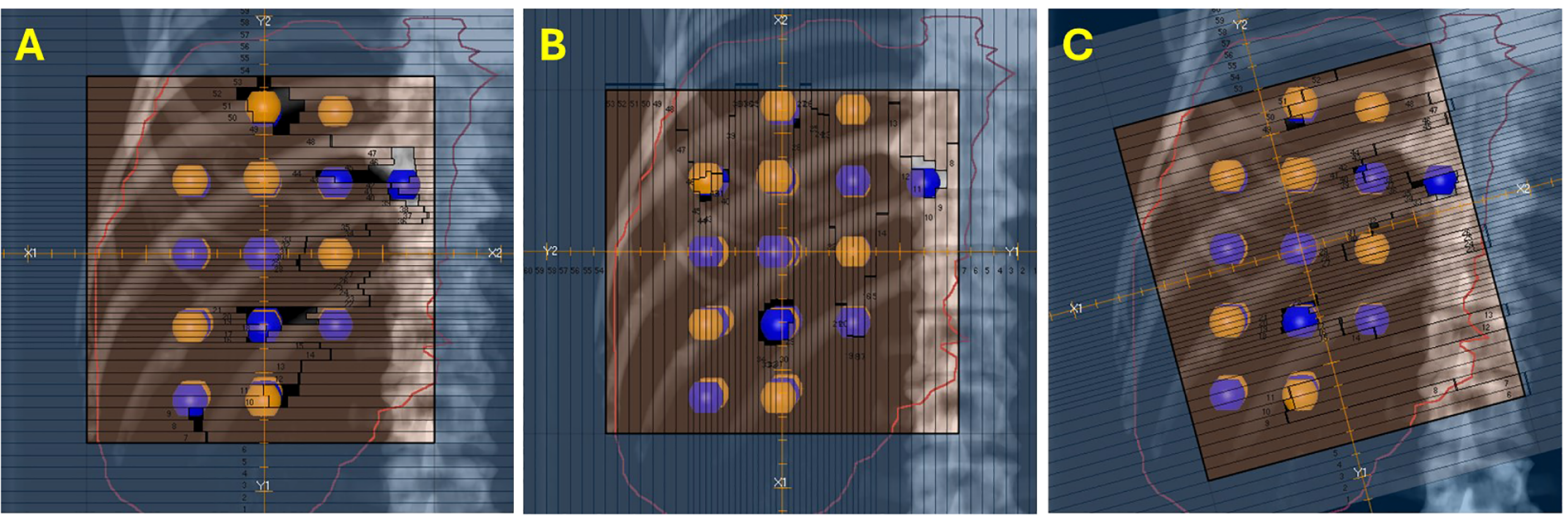
Collimator angles for VMAT-LRT. Blue, high-dose spheres (VTVH); orange, low-dose spheres (VTVL). **(A, B)** Collimator angles of 0°, 90°, or 270° for four out of five full arcs to optimize the modulation between lattice spheres. **(C)** One 15° off beam was also utilized to modulate around the spheres for a more conformal dose distribution.

VMAT-LRT plans were created following clinical workflow, using the RayStation treatment planning system (version 12A; RaySearch Laboratories, Stockholm, Sweden) and 5 ± 1 flattening-filter-free 6-MV full arcs. Plans were generated on a TrueBeam linear accelerator (Varian Medical Systems, Palo Alto, CA) and dose calculations were performed with the collapsed cone algorithm. Collimator angles were chosen on the basis of the geometry of lattice spheres (**Figure 2**). Two arcs had collimator angles of 0°, two arcs had collimator angles of 90° or 270°, and one arc had a collimator angle of 15° off (15°, 315°, 30°, or 45°).

The majority of the VMAT planning structures were created using the same in-house custom script for consistency and efficiency, including fall-off rings (2–3 consecutive 5 mm rings) for the VTVH and VTVH, and structures to control max dose between sphere layers, and to the GTV periphery and normal tissue. Some cases required generating planning structures to control max dose to nearby OARs or hot or cold spots.

#### 2.2.2 RAD-LRT

**Figure 3:**
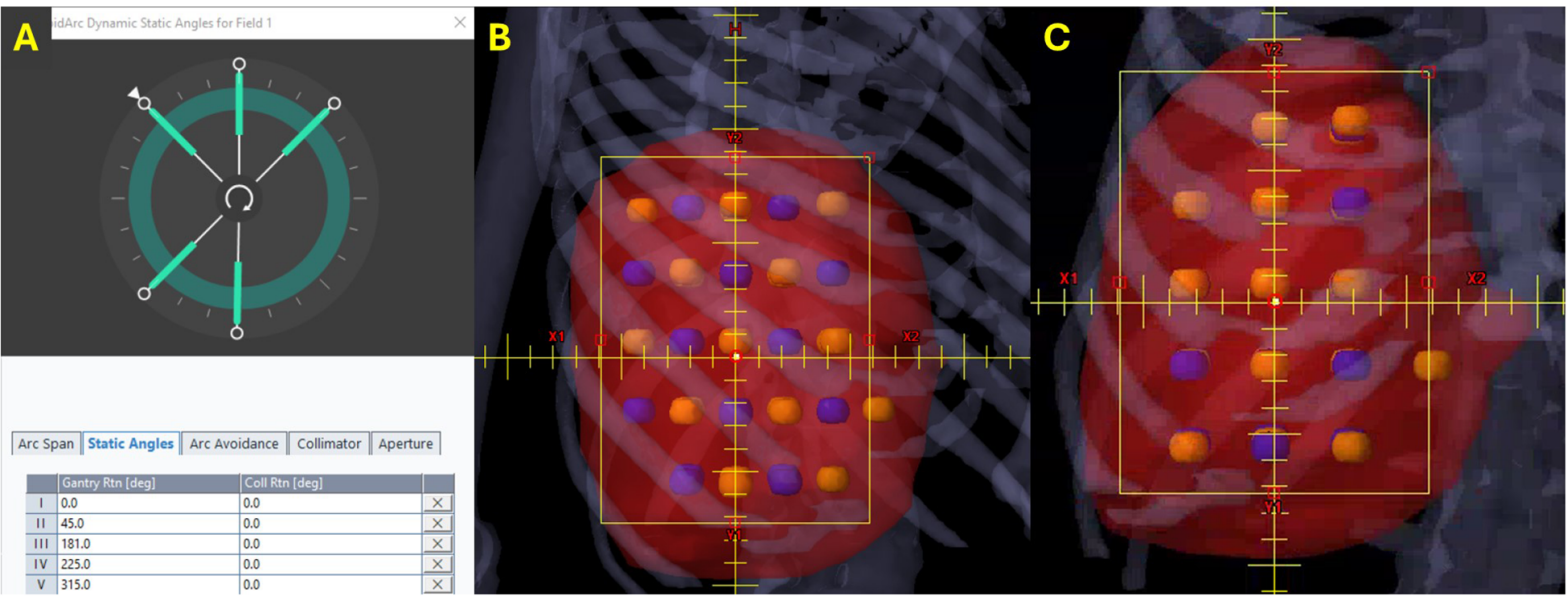
Static angles for RAD-LRT. Blue, high-dose spheres (VTVH); orange, low-dose spheres (VTVL). **(A)** All RAD cases were initially optimized with the same 5 static angles. **(B)** Three angles that capitalize on overlapping VTVH spheres without intersecting VTVL spheres (beams eye view). **(C)** Two angles that still modulate along the lattice but have VTVH and VTVL overlap (beams eye view).

RAD-LRT plans were generated with the Eclipse treatment planning system, version 18.1 (Varian Medical Systems, Palo Alto, CA, USA), using a single flattening-filter-free 6-MV full arc. Plans were generated on a TrueBeam linear accelerator (Varian Medical Systems, Palo Alto, CA) and calculated with the Acuros XB algorithm, version 18.1.0. RAD-LRT plans were generated using the same target and OAR structures used to generate the VMAT-LRT plans. RAD introduces a dynamic collimator, along with the ability to optimize collimator rotation throughout the arc; this feature was selected for the arc modulation between static fields. These static fields serve as ports, where the gantry rotation is paused during arc delivery to allow for more focused modulation, akin to IMRT fields. Applying the same logic as the VMAT-LRT plans, the static angles had a fixed 0° collimation to ensure modulation along the lattice (**Figure 2A**). Static angles were selected according to tumor location (proximity to skin surface or OARs) and sphere placement using the beams eye view (**Figure 3**). For all cases, the same 5 static angles (0°, 45°, 181°, 225°, and 315°) were initially chosen, and then adjustments were made for each case (e.g., removed 181° for medial tumors to avoid the spinal cord, replaced 45° with 135° for right posterior targets) (**Figure 3**). The RAD optimizer allows for adjusting preference for modulation weighting between the arc sections and static angles, which are simultaneously optimized (24). All RAD-LRT cases were completed with balanced weighting between arc and static fields, which corresponds to 26 control points per port.

RAD-LRT plans were completed using a template, which included the following planning structures: normal tissue control, VTVL 5 mm expansion, VTVH 1.5 cm expansion, and a structure to control dose between sphere layers. Some cases required generating planning structures to control max dose to nearby OARs. Contouring hot or cold spots were not obviously necessary or beneficial for these cases, potentially due to RAD restarting optimization between runs (i.e., does not allow continuation of previous optimization) (24).

#### 2.2.3 Plan objectives

The primary objective was delivery of 20 Gy in 1 fraction to 80% of the VTVH (the prescription dose); the secondary objective was delivery of a mean dose not exceeding 4 Gy to the VTVL (**Table 2**). All lattice spheres were also kept as separate contours in the treatment planning system to allow for dose-volume histogram (DVH) visualization of each sphere to ensure adequate target coverage and minimal dose to the VTVL. Dose constraints for OARs, including the uninvolved liver, kidneys, stomach, duodenum, heart, and spinal cord, were based on institutional protocols or guidelines (**Table 3**).

**Table 2:** Lattice RT target volume objectives and constraints.

Target volume	Target objectives	Constraint
GTV	D0.03cc< 25Gy	D0.03cc< 28Gy
VTVH	D80% ≥ 20Gy	D80% ≥ 20Gy
	D90% ≥ 18Gy	
VTVL	D_mean_ ≤ 4Gy	D_mean_ ≤ 5Gy

GTV, gross tumor volume; VTVH, vertex tumor volume high; VTVL, vertex tumor volume low.

**Table 3:** Lattice RT OAR objectives and constraints.

Structure	OAR objectives	Constraint
Spinal cord	D0.03cc< 6 Gy	D0.03cc< 6 Gy
Esophagus	D0.03cc< 6 Gy	ALARA
Heart	D0.03cc< 6 Gy	ALARA
Stomach	D0.03cc< 6 Gy	ALARA
Duodenum	D0.03cc< 6 Gy	ALARA
Rt kidney	V7Gy< 67%	ALARA
Lt kidney	V7Gy< 67%	ALARA
Kidneys	D_mean_< 7 Gy	D_mean_< 12.50 Gy
Liver-GTV	D700cc (ALARA)	V20Gy< 700 cc
Small bowel	D0.03cc< 6 Gy	ALARA
Large bowel	D0.03cc< 6 Gy	ALARA
Rectum	D0.03cc< 6 Gy	ALARA
Bladder	D0.03cc< 6 Gy	ALARA

ALARA, as low as reasonably achievable; D700cc, maximum dose delivered to 700 cc of the target volume; V7Gy, volume receiving at least 7 Gy; V20Gy, volume receiving at least 20 Gy.

### 2.3 Plan review and statistical analyses

All final plans were reviewed by a dosimetrist and a medical physicist, both knowledgeable in VMAT-LRT and RAD-LRT planning. The peak volume (VTVH) was defined as the concatenation of all high-dose vertices. The valley volume (VTVL) was defined as the concatenation of all low-dose vertices. The maximum doses delivered to 5%, 10%, 20%, 50%, 80%, 90%, 95%, and 100% of the target volume (D5%, D10%, D20%, D50%, D80%, D90%, D95%, and D100%, respectively), along with the mean dose and max dose (D0.03cc), were assessed for VTVH and VTVL for all VMAT-LRT and RAD-LRT plans. The authors proposed and evaluated a surrogate equation to calculate peak-to-valley dose ratios (PVDRs). As the goal is to evaluate the overall target dose heterogeneity, the ratio of the near max (D90%) of the peak (VTVH) dose and the near min (D90%) of the valley (VTVL) dose was selected for the definition of PVDR for this study.

PVDR was defined by the following formula:


$$PVDR=\frac{\text{D}90\%\text{ VTVH}}{\text{D}90\%\text{ VTVL}}\;$$

The max dose was recorded for the tissue 1.0 cm outside of the GTV and for the PRV_LRT_ to assess normal tissue sparing. A paired Wilcoxon signed-rank test was used for comparison of all LRT dose metrics, with statistical significance determined using a Bonferroni-adjusted threshold of p< 0.0016 (0.05/31 comparisons) to reduce the risk of Type I errors for multiple comparisons. All statistical analyses were performed using RStudio (version 4.2.1; Posit Software; Boston, Massachusetts).

In addition to being evaluated in terms of dose statistics, each treatment planning approach was evaluated in terms of efficiency in treatment planning, specifically planning time (i.e., optimization time and additional planning structure generation (structures not generated by in-house script)), and number of beams and planning structures.

## 3 Results

### 3.1 Dose statistics

**Figure 4:**
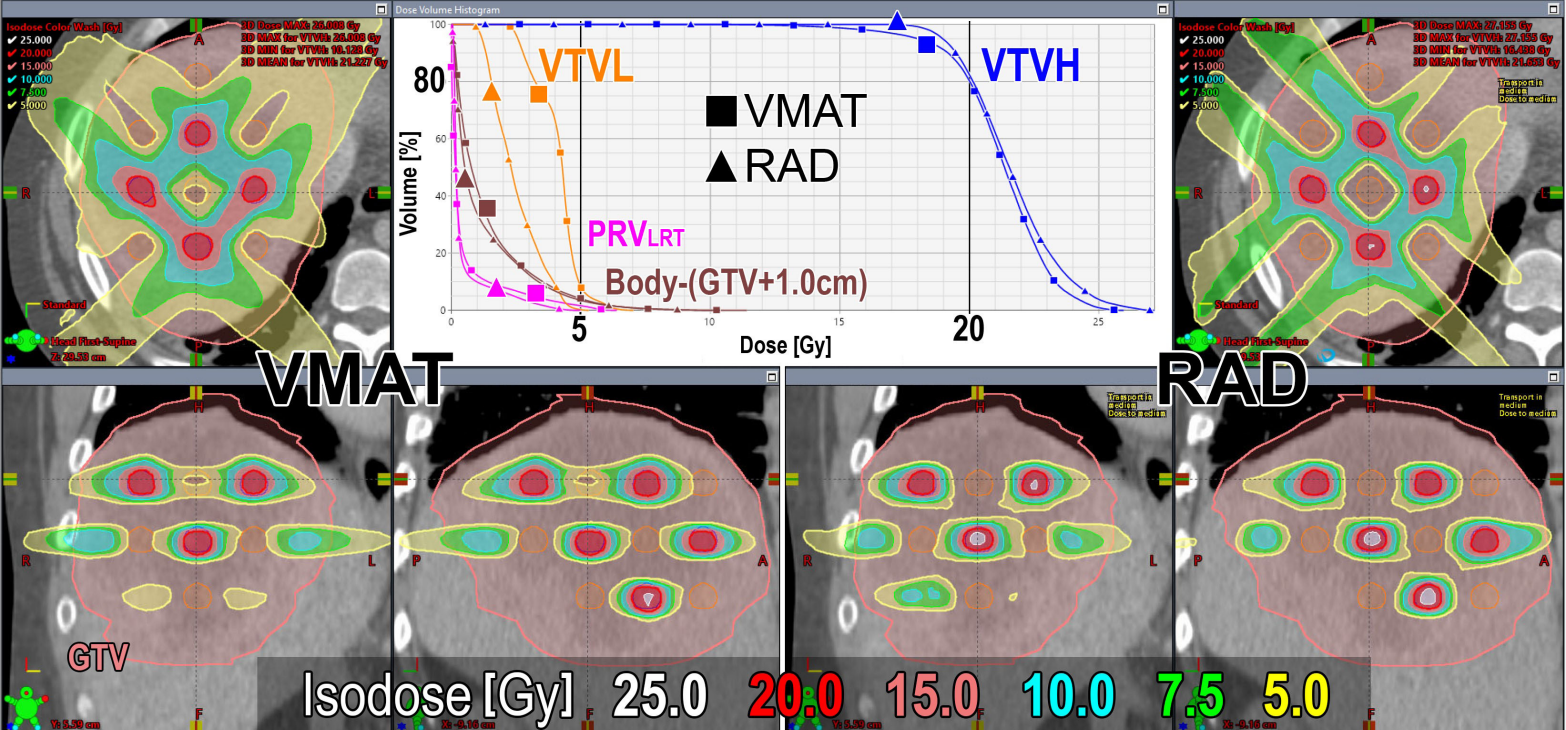
Dose distributions and DVH comparison of VMAT (left/square) and RAD (right/triangle) plans for case 4 (case details in **Table 1**). GTV is contoured in pink, VTVL (low-dose spheres) is contoured in orange, and VTVH (high-dose spheres) is contoured in blue. Prescription is D80% ≥20 Gy in 1 fraction.

**Figure 4** shows a representative comparison of the dose distributions and DVH for the VMAT-LRT and RAD-LRT plans, for case 4 (see **Table 1** for case details). The figure demonstrates the benefit of RAD in minimizing 5 Gy to centroid VTVL spheres without reduction in surrounding VTVH coverage or conformality.

**Figure 5:**
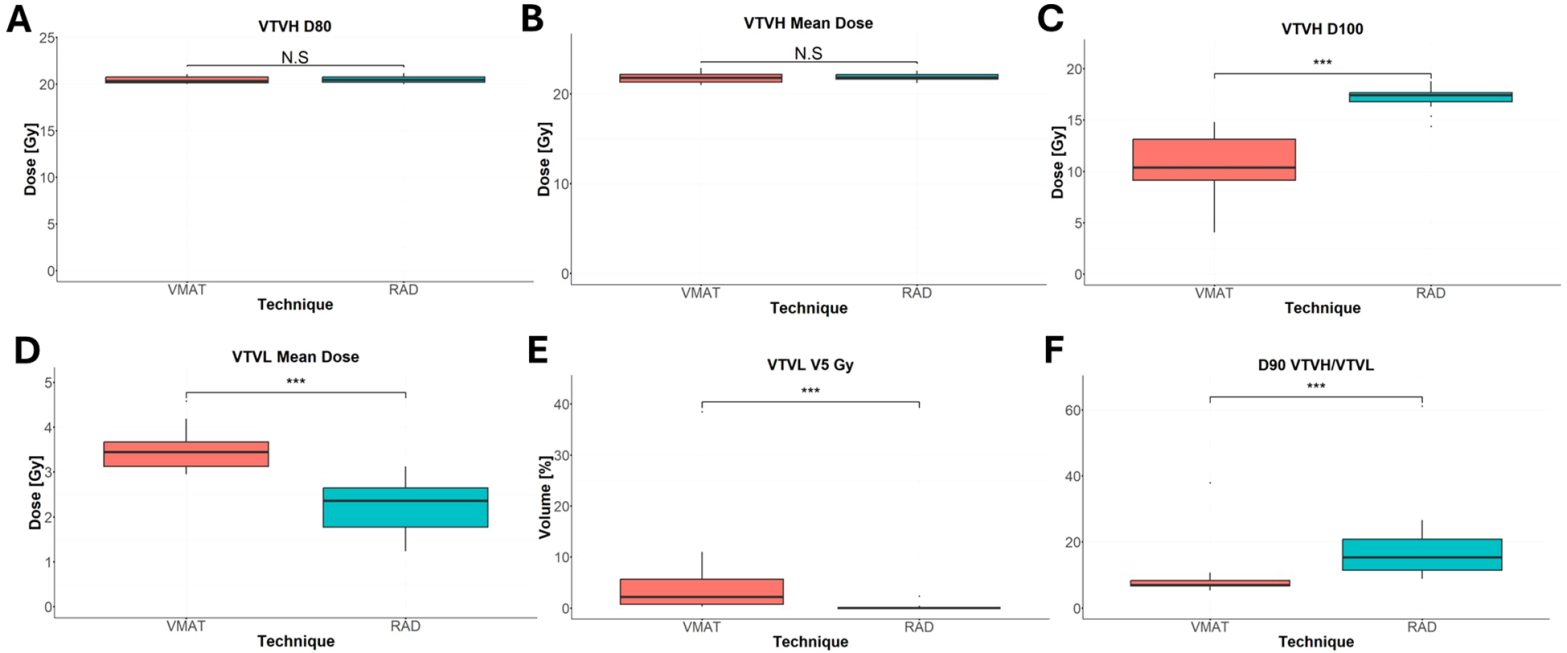
Comparison of VTVH and VTVL dose statistics between RAD-LRT and VMAT-LRT. **(A)** VTVH D80%. **(B)** VTVH mean dose. **(C)** VTVH D100%. **(D)** VTVL mean dose. **(E)** VTVL V5 Gy. **(F)** D90 VTVH/VTVL. N.S., not significant; ***, p<0.0001.

**Figure 6:**
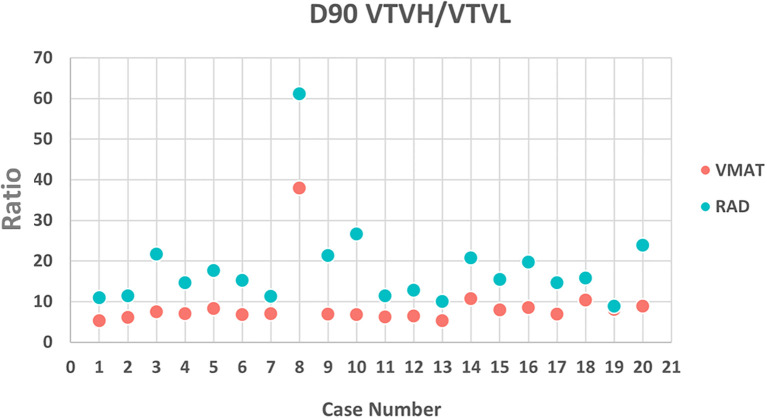
PVDR calculated as (D90% VTVH)/(D90% VTVL) for VMAT and RAD for each case. PVDR was higher in the RAD plan for each case, signifying a larger achievable difference between the peaks and valleys.

All VMAT-LRT and RAD-LRT plans met the OAR constraints and target objectives (**Table 4**). VMAT-LRT and RAD-LRT plans met the target prescription of D80% ≥20 Gy (**Table 4**, **Figure 5A, B**); however, RAD-LRT plans had significantly higher D90%, D95%, and D100% values, indicating higher target conformality (**Table 4**, **Figure 5C**). Across all DVH statistics, the VTVL doses were significantly lower with RAD-LRT than with VMAT-LRT (**Table 4**, **Figure 5D, E**). PVDRs were significantly higher for RAD-LRT (**Table 4**, **Figure 5F**), when compared to VMAT_LRT, indicating higher achieved dose heterogeneity. **Figure 6** shows PVDR achieved for each case’s VMAT-LRT and RAD-LRT plans.

**Table 4:** Dose-volume histogram metrics for VMAT and RAD.

Metric	Median (range) [Gy]	VMAT group mean [Gy]	RAD group mean [Gy]	P-value
VTVH				
Mean Dose	21.83 (20.98-23.44)	21.70	21.90	0.16
D5%	24.69 (23.46-26.54)	24.80	24.50	0.09
D10%	24.14 (23.00-25.81)	24.20	24.00	0.11
D20%	23.44 (22.37-25.10)	23.50	23.30	0.08
D50%	21.92 (21.12-23.36)	22.00	23.30	0.21
D80%	20.48 (20.00-21.87)	20.40	20.50	0.38
D90%	19.41 (11.76-21.32)	18.80	20.00	<0.0001*
D95%	18.29 (8.29-20.88)	17.00	19.50	<0.0001*
D100%	13.89 (4.05-18.77)	10.60	17.10	<0.0001*
D0.03cc	25.77 (24.30-27.38)	25.90	25.60	0.14
VTVL				
Mean Dose	2.86 (1.23-4.58)	3.40	2.20	<0.0001*
D5%	4.20 (2.14-7.47)	4.80	3.50	<0.0001*
D10%	3.87 (1.96-6.13)	4.40	3.30	<0.0001*
D20%	3.52 (1.63-5.78)	4.10	2.90	<0.0001*
D50%	2.80 (1.09-4.42)	3.40	2.20	<0.0001*
D80%	2.16 (0.41-3.68)	2.70	1.60	<0.0001*
D90%	1.90 (0.32-3.30)	2.40	1.30	<0.0001*
D95%	1.71 (0.28-3.08)	2.20	1.10	<0.0001*
D100%	1.23 (0.21-2.33)	1.60	0.80	<0.0001*
D0.03cc	5.00 (2.50-9.18)	5.80	4.10	<0.0001*
V5 Gy	2.50% (0.00%-38.00%)	5.00%	0.15%	<0.0001*
Body-(GTV + 1.0cm)				
D0.03cc	8.57 (5.64-10.85)	8.20	8.90	0.98
PRV_LRT_				
D0.03cc	8.18 (3.35-20.16)	9.67	6.70	<0.0001*
VTVH/VTVL				
D50%	9.12 (4.79-31.19)	6.52	11.72	<0.0001*
D80%	12.09 (5.47-49.24)	8.57	15.61	<0.0001*
D90%	13.58 (5.31-61.06)	8.92	18.20	<0.0001*
D100%	16.57 (1.73-78.04)	7.77	25.37	<0.0001*
Liver minus GTV				
Mean Dose	1.17 (0.5-2.43)	1.21	1.13	<0.05
D700cc	0.70 (0-2.25)	0.77	0.64	<0.001
D0.03cc	9.14 (6.47-13.88)	9.40	8.88	0.18
Max Dose	9.63 (6.88-14.75)	9.95	9.30	0.10

VTVH, vertex tumor volume high; VTVL, vertex tumor volume low; PRV_LRT_, GI luminal contours with a 1.0–1.5 cm (GTV<1000 and GTV≥1000 respectively) expansion; VMAT, volumetric modulated arc therapy; RAD, rapid arc dynamic; Body-(GTV + 1.0cm), Body contour minus GTV with a 1.0 cm margin.

*p<0.0016 – Statistical significance determined using a Bonferroni-adjusted threshold.

Sparing of the normal tissue 1.0 cm outside of the GTV was similar for VMAT-LRT and RAD-LRT; however, the PRV_LRT_ received a significantly lower maximum dose in the RAD-LRT plans (**Table 4**). VMAT-LRT and RAD-LRT produced similar levels of sparing of the liver minus GTV, although RAD-LRT showed a slight advantage.

### 3.2 Planning efficiency

RAD was generally efficient for creating quality LRT plans. RAD-LRT plans required fewer planning structures (9 ± 1 for VMAT-LRT, 4 ± 1 for RAD-LRT), less planning time (26 ± 8 min for VMAT-LRT, 18 ± 11 min for RAD-LRT), and fewer treatment beams (5 ± 1 arcs for VMAT-LRT, 1 arc with 4 ± 1 static ports for RAD-LRT). In addition, all RAD-LRT cases followed the same planning template and steps, with only 2 plans requiring further editing of optimization objectives, suggesting that RAD-LRT requires minimal customization between cases.

## 4 Discussion

This study showed that there is a significant advantage to using RAD over standard VMAT for LRT, which is a widely adopted SFRT approach. The novel components of RAD, specifically the optimized dynamic collimator rotation and increased modulation at strategically selected static gantry angles, mitigate many of the inherent complexities traditionally associated with treatment planning for LRT [i.e., the required dose heterogeneity to treat target with high enough cancer-killing dose while also achieving low enough valley dose to spare channels of tissue between target spheres (7, 16, 17)]. This is the first study to compare the dosimetry of RAD and VMAT for LRT treatment planning.

The advantages of utilizing RAD for LRT were observed across a wide array of bulky liver tumors with a dataset of GTVs ranging from 552 cm^3^ (16% of the whole liver) to 2,578 cm^3^ (85% of the whole liver) (**Tables 1**, **4**). This range of GTVs allowed for comprehensive testing and comparison, in line with previous studies (7, 22). Primarily, RAD appears to be better able to modulate the dose between separated targets, thus allowing for both more conformal VTVH (high-dose/peak spheres) and significantly reduced dose to the VTVL (low-dose/valley spheres) (**Table 4**, **Figure 5**). In LRT, reducing valley doses below 5 Gy is desirable if the treatment goal is tumor debulking through synergy of tumor-killing and immunomodulatory effects, as a dose below 5 Gy retains adequate perfusion of immune cells (4, 7, 22, 25, 26). The mean proportion of the VTVL receiving ≤5 Gy was 5% for VMAT but just 0.1% for RAD (**Table 4**). Rivera et al. (27) and Fernandez-Palomo et al. (28) both showed that in SFRT, the correlation between valley minimum and treatment response was stronger than the correlation between peak dose and treatment response, which emphasizes the importance of the significantly lower doses to the VTVL that RAD is able to achieve. The use of VTVL spheres, which were systematically placed along the lattice between the VTVH spheres, ensured even distribution of channels of intact perfusion throughout the tumor while maintaining the global geometric structure (18).

Of note, the lattice and vertices were automatically generated using an in-house script, which standardized the LRT setup and planning approach for all cases. Zhang et al. emphasized the importance of standardization of LRT not only for patient care but also for comparability of clinical trials and outcome results (5). Tucker et al. (29) and Gaudreault et al. (30) showed promising results with script-based algorithms to generate LRT lattice structures; both groups showed reduced dependency on user experience for sphere placement and hands-on planning time. Gaudreault et al. found that a fixed lattice geometry was not suitable for all patients (31), and in their follow-up study, they found that optimal lattice geometry can be estimated on the basis of tumor volume (30). As such, our script was designed with the option of generating lattice geometry for tumor volumes smaller than 1,000 cm^3^ or 1,000 cm^3^ or larger (details in Section 2.1), and can be further customized for variable lattice size or CTC distance.

While there is a general consensus on the need to evaluate the PVDR for LRT plans, exactly how to calculate this metric has been an ongoing topic of discussion (5, 21, 32, 33). **Supplementary Figure S1** depicts VMAT-LRT and RAD-LRT comparison using other common PVDR definitions. Since our goal was to systematically minimize valley dose between peaks, we incorporated low-dose valley spheres as avoidance structures rather than a “GTV minus high-dose spheres” volume. Given this approach, we utilized a PVDR surrogate metric of (D90% VTVH)/(D90% VTVL). This summary metric is in line with the theory behind the Radiosurgery Society white paper recommendation to use D90/D10 (i.e., VPDR) as single point doses may not accurately represent plan quality (5). The RAD plans in our study had significantly higher PVDR than the VMAT plans had, signifying that RAD was able to achieve a larger difference between the peaks and valleys, with the bulk of the difference due to the lower VTVL dose (**Table 4**, **Figures 5**, **6**).

Although proton and carbon ion therapies have also shown lower valley doses than VMAT (34, 35), photon RT is more widely accessible. RAD offers an alternative form of photon radiation delivery through software update (Eclipse version 18.1; Varian Medical Systems, Palo Alto, CA) and utilization of existing hardware (TrueBeam 4.1; Varian Medical Systems, Palo Alto, CA) and therefore is more readily accessible than proton or heavy-ion SFRT at this time.

A limitation of this study is that while we have access to the planning portion of RAD treatment, we do not currently have a method to validate through machine delivery and measurement, so it was not possible to determine if these plans are deliverable as currently created. Continued research on validation and deliverability of these plans would be interesting, as well as further exploration of other treatment sites. As the plans were completed in different treatment planning systems that utilize different calculation algorithms, further research should be done to validate results using the same system. However, it should be noted that both VMAT-LRT and RAD-LRT plans used currently standard of care treatment planning systems and the most common clinically utilized dose calculation algorithms. Another limitation is that it is difficult to directly compare the results of this study with results of other studies, as there is little standardization in lattice treatment and plan metric reporting at this time (7).

Lastly, for almost all RAD plans, the planning goals were met with use of only a planning template, and only 2 plans required customization, suggesting that less experience may be required to generate a quality RAD-LRT plan than to generate a quality VMAT-LRT plan. Further research into standardization through automated plan setup and treatment planning could be useful as LRT becomes more mainstream.

## 5 Conclusion

In this study, an LRT planning approach utilizing a custom in-house script to generate the lattice spheres was used to generate plans for 2 photon dose delivery methods, VMAT and RAD. RAD-LRT showed high plan quality, as indicated by comparable target coverage but lower valley doses when compared to conventional VMAT-LRT treatment plans. RAD-LRT also demonstrated high treatment planning efficiency, suggesting that RAD may offer a unique approach to planning this complex SFRT modality.

## Data Availability

The raw data supporting the conclusions of this work will be made available upon reasonable request and commensurate with a valid data sharing agreement with MD Anderson Cancer Center.
