# Supplementary material for: Novel volumetric modulated arc therapy approach for lattice radiation therapy for bulky liver tumors
Source: Front Oncol. 2025 Oct 31;15:1680342. doi: 10.3389/fonc.2025.1680342 (PMC12615216; doi:10.3389/fonc.2025.1680342)

Novel Volumetric Modulated Arc Therapy Approach for Lattice Radiation Therapy for Bulky Liver Tumors

Christine V. Chung[1*], Saurabh S. Nair[1], Meena S. Khan[1], Callistus I. Nguyen[1], Rachael M. Martin-Paulpeter[1], Ethan Ludmir[2], Laurence E. Court[1], Joshua S. Niedzielski[1*]

[1]Department of Radiation Physics, The University of Texas MD Anderson Cancer Center, Houston, TX, United States
[2]Department of Gastrointestinal Radiation Oncology, The University of Texas MD Anderson Cancer Center, Houston, TX, United States

***Corresponding Authors:**
Joshua Niedzielski, jsniedzielski@mdanderson.org; Christine Chung, cchung2@mdanderson.org

Introduction

This document is the supplementary material to the article "Novel Volumetric Modulated Arc Therapy Approach for Lattice Radiation Therapy for Bulky Liver Tumors." The paper investigated the feasibility and dosimetric implications of employing RapidArc Dynamic (RAD) for the delivery of lattice radiation therapy (LRT). Specifically, it compared RAD-LRT with VMAT-LRT in terms of fulfillment of plan objectives, normal tissue sparing, and the efficiency of treatment planning. It concluded that RAD may offer a unique approach to LRT. RAD-LRT was shown to generate high quality plans with notable treatment planning efficiency, allowing for creation of quality plans without extensive planning time and LRT expertise.

The study used a surrogate marker for peak-to-valley-dose-ratio (PVDR) evaluation, which measures the target heterogeneity between the peak target dose and the valley low dose. There are multiple varying equations in the literature for PVDR. This supplementary material serves to display VMAT-LRT vs RAD-LRT results using several other PVDR definitions.

List of Abbreviations

D10	Dose at 10% volume	RAD	RapidArc Dynamic
D90	Dose at 90% volume	VMAT	Volumetric modulated arc therapy
Dmean	Mean dose	VPDR	Valley-to-peak dose ratio
GTV	Gross tumor volume	VTVH	Vertex tumor volume high, analogous to the peak dose
PVDR	Peak-to-valley dose ratio	VTVL	Vertex tumor volume low, analogous to the valley dose

References

1. Bhagyalakshmi AT, Ramasubramanian V. Assessing dosimetric advancements in spatially fractionated radiotherapy: From grids to lattices. Med Dosim [Internet]. 2024 [cited 2024 Nov 25];49(3):206–14. Available from: https://linkinghub.elsevier.com/retrieve/pii/S0958394723001164
2. Bhagyalakshmi AT, Ramasubramanian V. Impact of Number and Placement of High-dose Vertices on Equivalent Uniform Dose and Peak-to-valley Ratio for Lattice Radiotherapy. J Med Phys [Internet]. 2024 [cited 2025 June 23];49(4):493–501. Available from: https://www.ncbi.nlm.nih.gov/pmc/articles/PMC11801099/

SUPPLEMENTARY MATERIAL

Figure S1. Comparison of VMAT-LRT and RAD-LRT dose statistics for varying PVDR equations. PVDR1 is used in the main body of the paper. PVDR2 is another surrogate metric initially proposed by authors. PVDR3-5 and VPDR1 are outlined and referenced in Bhagyalakshmi's and Ramasubramanian's work. **, p<0.001; ***, p<0.0001.

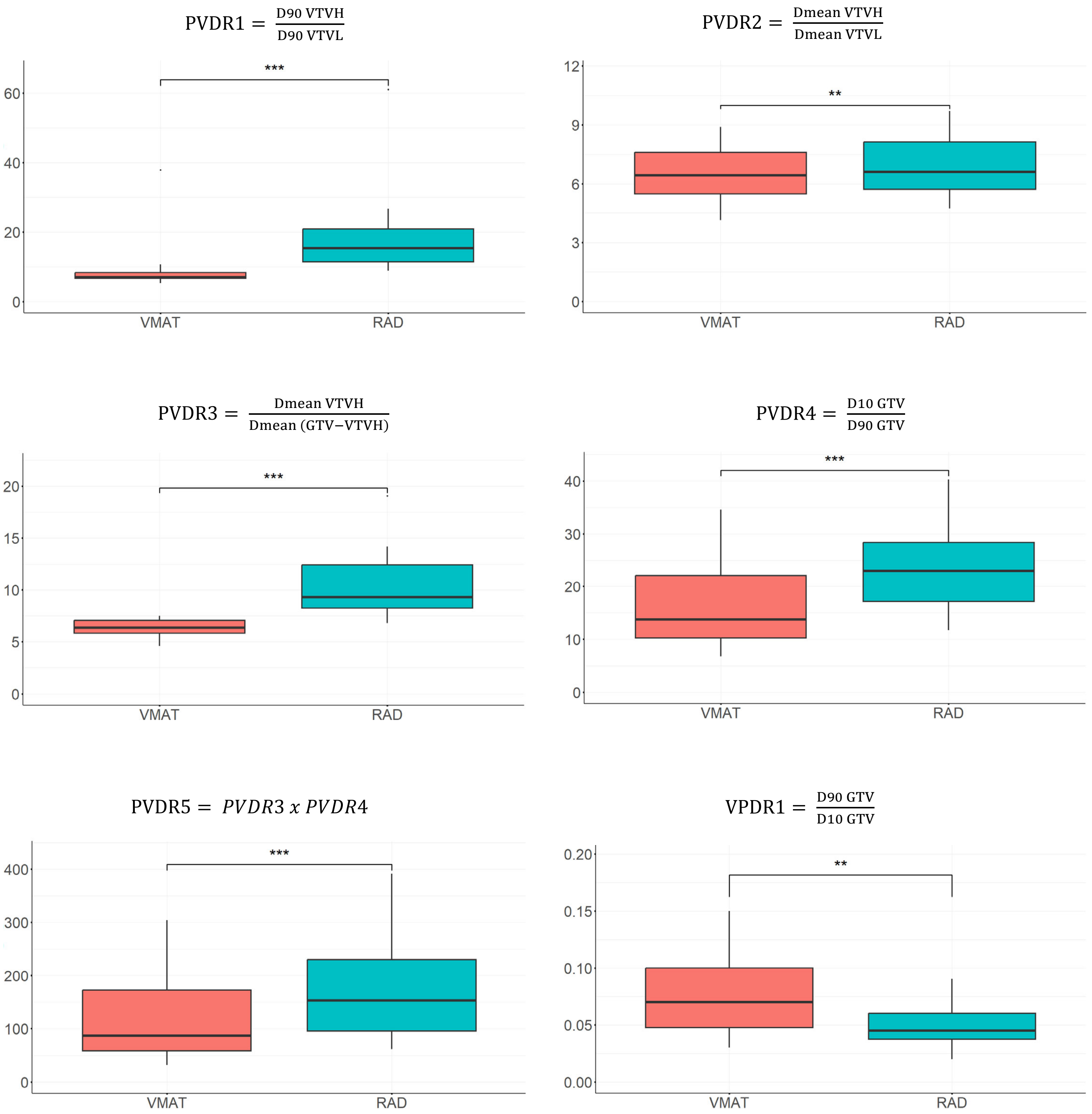

Supplement: Supplementary file 1 [file DataSheet1.pdf]
